# Trends and outcomes of women with synchronous endometrial and ovarian cancer

**DOI:** 10.18632/oncotarget.25550

**Published:** 2018-06-19

**Authors:** Koji Matsuo, Hiroko Machida, Erin A. Blake, Laura L. Holman, Bobbie J. Rimel, Lynda D. Roman, Jason D. Wright

**Affiliations:** ^1^ Division of Gynecologic Oncology, Department of Obstetrics and Gynecology, University of Southern California, Los Angeles, CA, USA; ^2^ Norris Comprehensive Cancer Center, University of Southern California, Los Angeles, CA, USA; ^3^ Division of Gynecologic Oncology, Department of Obstetrics and Gynecology, University of Oklahoma Health Sciences Center, Oklahoma City, OK, USA; ^4^ Division of Gynecologic Oncology, Cedars-Sinai Medical Center, Los Angeles, CA, USA; ^5^ Division of Gynecologic Oncology, Department of Obstetrics and Gynecology, Columbia University College of Physicians and Surgeons, New York, NY, USA

**Keywords:** endometrial cancer, ovarian cancer, trend, survival, synchronous

## Abstract

This retrospective observational study examined trends, characteristics, and survival of women with synchronous endometrial and ovarian cancer (SEOC) in the Surveillance, Epidemiology, and End Results Program between 1973 and 2013. Among 235,454 women with primary endometrial cancer, synchronous ovarian cancer was seen in 4,082 (1.7%) women with the proportion being decreased from 2.0% to 1.6% between 1983 and 2013 (*P*=0.049); and the proportion of concurrent endometrioid tumors in the two cancer sites has increased from 24.2% to 49.9% among SEOC women (*P*<0.001). When compared to endometrial cancer without synchronous ovarian cancer, endometrioid histology in the two cancer sites was associated with improved cause-specific survival while non-endometrioid histology in the ovarian cancer was associated with decreased cause-specific survival (adjusted-*P*<0.01). Among 110,063 women with primary epithelial ovarian cancer, synchronous endometrial cancer was seen in 3,940 (3.6%) women with the proportion being increased from 2.2% to 4.4% between 1973 and 2013 (*P*<0.001); and the proportion of concurrent endometrioid tumors in the two cancer sites had increased from 24.3% to 50.2% among SEOC women (*P*<0.001). When compared to primary epithelial ovarian cancer without synchronous endometrial cancer, SEOC was associated with better cause-specific survival if ovarian cancer is endometrioid type or if endometrial cancer is endometrioid type (adjusted-*P*<0.001). Across the two cohorts, the proportion of SEOC reached to the peak in the late-40 years of age and then decreased significantly (*P*<0.001). In conclusion, our study suggests that synchronous ovarian cancer has decreased among endometrial cancer whereas synchronous endometrial cancer has increased among epithelial ovarian cancer.

## INTRODUCTION

Synchronous endometrial and ovarian cancer (SEOC) is defined as the simultaneous presence of these two cancers at the time of diagnosis as opposed to metachronous cancer where these two cancers are diagnosed at different chronologic time points. SEOC is not a rare clinical entity; it has been reported in 3-10% of ovarian cancers and 3-5% of endometrial cancers [1–4]. The substantial range of incidences for synchronous endometrial cancer in ovarian cancer may be due to the small sample sizes in the majority of prior analyses. In addition, population-based statistics are missing regarding the incidence of synchronous ovarian cancer in endometrial cancer.

The diagnosis of SEOC is generally made by histopathologic evaluation. The landmark criteria for the diagnosis of SEOC were established by Ulbright and Roth in 1985, and more solid criteria were endorsed by Scully *et al* in 1998 [5–7]. To date, the impact of these diagnostic criteria on the incidence trend of SEOC is not known. Historically, women with SEOC, specifically those who have concordant endometrioid tumors in the two cancer sites, have been thought to have a favorable prognosis [1, 7]. Because prior studies evaluating prognosis have lacked a control group of endometrial cancer without synchronous ovarian cancer, the histologic pattern-specific survival of women with synchronous tumors relative to those without synchronous tumors will be useful to understand the clinical characteristics of this disease entity.

In the past decades, the demographics of endometrial cancer and ovarian cancer have been changing in the United States. There has been a gradual increase in incidence of endometrial cancer most likely due to the significant increase in obesity [8, 9]. Moreover, there is a steady decrease in incidence of ovarian cancer likely due to the introduction of oral contraceptive use and reduction in menopausal hormone therapy [10–12]. Given these recent changes in demographics, it is of interest to understand the time-trends of SEOC in the United States. The objective of the study was to examine population-based trends, characteristics, and survival outcome of women with SEOC.

## PATIENTS AND METHODS

### Data source and eligibility

This retrospective study utilized the Surveillance, Epidemiology, and End Results (SEER) Program that is a population-based tumor registry in the United States [13]. This database was provided and has been maintained by the National Cancer Institute since 1973. The SEER database covers approximately 28% of the US population and is publicly available and de-identified. The data entry to this database is performed by staff personnel who are trained by the National Cancer Registrars Association with rigorous quality control [14]. The Institutional Review Board at the University of Southern California exempted this study. The STROBE guidelines were used to outline the performance of this observational study [15].

SEER^*^Stat 8.3.2 (IMS Inc., Calverton, MD, USA) was used to extract the SEER18 cases (1973-2013), generating the dataset from “Corpus Uteri / Uterus NOS” limited to malignancy and female sex. Primary endometrial cancer cases were eligible for the study, excluding sarcoma or metastatic tumors to the uterus from another origin. SEER^*^Stat 8.3.2 was also used to generate primary ovarian cancer cases for the same study period. Then, the ovarian cancer dataset was linked with the aforementioned endometrial cancer dataset. The same study identification numbers between the two datasets were considered secondary primary cancer in the same individual as described previously [16–18]. This methodological approach was based on the rationale that the SEER Program maintains the records per cancer type but not an individual basis.

The chronologic time sequence of the endometrial cancer diagnosis date and the ovarian cancer diagnosis date were then examined among the cases recorded in the two datasets. The cohort cancer was used as the index cancer whereas the non-cohort cancer represented the secondary primary cancer to determine this time interval for each cohort. Women in whom the time intervals between the two diagnoses were less than 4 months were considered synchronous ovarian cancer in the endometrial cancer cohort. The cutoff value of a 4-month time interval between the two cancer diagnoses is based on the rationale that endometrial cancer is commonly diagnosed *via* endometrial sampling prior to hysterectomy and ovarian cancer is generally diagnosed at the time of subsequent hysterectomy. Waiting time for hysterectomy-based surgery after endometrial biopsy in women with endometrial cancer commonly reflects time interval for patient referral to specialty and optimization of medical condition as women with endometrial cancer often possess multiple comorbidities. The vast majority of women with endometrial cancer undergo hysterectomy within 4 months of diagnosis [19–21].

A similar approach was performed to generate the epithelial ovarian cancer cohort, and the dataset was linked to the endometrial cancer dataset to identify the secondary primary cancer cases. The ovarian cancer cohort did not include non-epithelial histologies. We also used the 4-month cutoff to define the synchronous endometrial cancer cases in the epithelial ovarian cancer cohort to be consistent with the endometrial cancer cohort. Time interval analyses between the two cancer diagnoses among secondary primary cancer cases relative to an endometrial cancer diagnosis showed that nearly half (54.5%) of secondary primary cancers were diagnosed at the same time and that the vast majority of cases (86.2%) were diagnosed within 4 months (Supplementary Figure 1).

### Clinical information

Among the eligible cases for analysis, patient demographics, tumor information, treatment patterns, and survival outcome were ascertained from the database. Patient demographics included age, year and month at diagnosis, ethnicity, marital status, and registration area. Tumor information included cancer stage, histologic subtype, tumor grade, and tumor size. Cause-specific survival (CSS), defined as the time interval between the date of cancer diagnosis and the date of death from the corresponding cancer, was examined for survival outcome. SEER Cause Specific Death Classification was utilized to determine CSS, and the code representing “death attributable to this cancer diagnosis” was used as a surrogate for CSS, and the code representing “death attributable causes other than this cancer diagnosis” was not used for CSS. Cause of death in this database is linked with the National Death Index and the state mortality records [22].

Recorded cancer stage was based on the American Joint Committee on Cancer 7th surgical-pathological staging classification schema [23]. The International Classification of Diseases for Oncology third edition site/histology validation and the World Health Organization histological classification codes were used for grouping histologic subtypes as reported previously (Supplementary Tables 1-2) [24]. Histologic types of endometrial and ovarian cancers were grouped as endometrioid and non-endometrioid, and the combination patterns of the two cancer sites were assessed in this study (Supplementary Table 3).

### Statistical consideration

The primary interest of analysis was to examine trends, characteristics, and survival of women with synchronous cancer in the two cohorts. The secondary interest of the analysis was to examine tumor characteristics and outcome based upon the histologic patterns of the two cancers. Continuous variables were assessed with Student *t* test or one-way ANOVA test as appropriate. Categorical and ordinal variables were assessed with chi-square test. On multivariable analysis, binary logistic regression models were used to assess the association of SEOC and clinico-pathological factors. Patient demographics and tumor characteristics were entered in the final model.

For trend analyses of SEOC per calendar year and age at diagnosis, Joinpoint Trend Software (version 4.4.0.0, National Cancer Institute, Bethesda, MD, USA) provided by the National Cancer Institute was used to determine the potential changes in temporal trends [25]. Time duration was grouped every one year or age to provide percent frequency of collected variables. The results were analyzed with linear segmented regression test, and log-transformation was performed to determine annual percent change (APC) of the slope with 95% confidence interval (CI) [26].

The Kaplan-Meier method was used to construct survival curves, and statistical difference between the curves were assessed with log-rank test for univariable analysis. Cox proportional hazard regression models were used to assess the independent association of SEOC and CSS on multivariable analysis. Patient demographics and tumor characteristics were entered in the final model. Magnitudes of statistical significance were expressed with adjusted-HR and 95%CI. All hypotheses were two-tailed, and a *P*-value of less than 0.05 was considered statistically significant. Statistical Package for Social Sciences (SPSS, version 24.0, IBM Corp, Armonk, NY, USA) was used for the analysis.

## RESULTS

### Endometrial cancer cohort

Among 246,736 cases of uterine cancer cases in the database, sarcoma (*n*=10,578), metastatic tumors to the uterus from malignancy of non-uterine origin (*n*=309), and multiple diagnosis cases (duplicated or second entry, *n*=395) were excluded. The remaining 235,454 cases represented primary endometrial cancer. Of those, endometrial cancer with synchronous ovarian cancer were seen in 4,082 (1.7%, 95%CI 1.7-1.8) cases.

**Figure 1:**
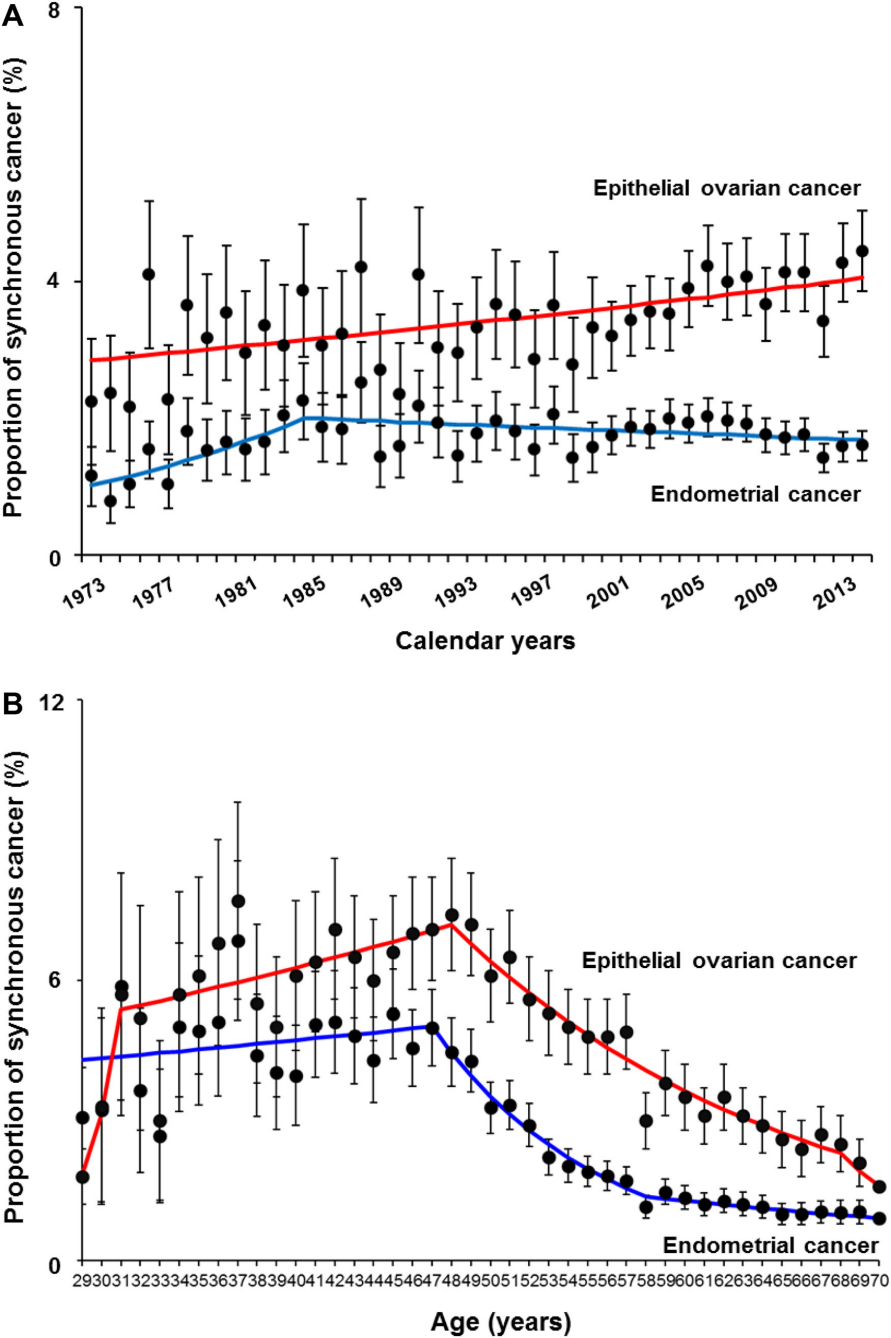
Trends of synchronous endometrial and ovarian cancer Proportion of synchronous cancer is shown per **(A)** calendar year and **(B)** age. Blue line depicts a trend of proportion of women with endometrial cancer who had synchronous ovarian cancer. Red line depicts a trend of proportion of women with epithelial ovarian cancer who had synchronous endometrial cancer. Dots represent percent proportion and error bars represent 95% confidence interval. Frequencies are shown in Supplementary Table 3.

Year trends of women with endometrial cancer who had synchronous ovarian cancer were examined (Figure 1A). The proportion of synchronous ovarian cancer significantly increased from 1.1% to 2.0% between 1973 and 1983 (APC 6.72, 95%CI 0.42-11.8, *P*=0.007) and then gradually decreased from 2.0% to 1.6% between 1983 and 2013 (APC -0.54, 95%CI -2.03 to -0.01, *P*=0.049). For age trends (Figure 1B), the proportion of synchronous ovarian cancer increased from 3.1% to 5.0% between 29 and 47 years of age, and then significantly decreased from 5.0% to 1.2% between 47 and 58 years of age (APC -11.0, 95%CI -12.8 to -9.3, *P*<0.001).

Characteristics of women with endometrial cancer who had synchronous ovarian cancer are shown in Table 1. Women with endometrial cancer who had synchronous ovarian cancer were more likely to be young and single and less likely to be of Black ethnicity (all, adjusted-*P*<0.05). Endometrial cancer with synchronous ovarian cancer was more likely to be stage I-II disease, endometrioid or serous histology types, grade 1-2 tumors, and small tumor size (all, adjusted-*P*<0.05).

**Table 1:** Patient demographics

Characteristic	Endometrial cancer cohort		Epithelial ovarian cancer cohort	
	Synchronous ovarian cancer (+)	Synchronous ovarian cancer (-)	Synchronous endometrial cancer (+)	Synchronous endometrial cancer (-)
Number	4,082 (1.7%)	231,372 (98.3%)	3,940 (3.6%)	106,123 (96.4%)
Age (y)	56.1 (±12.6)	63.5 (±12.4)	55.6 (±12.2)	62.3 (±14.3)
≥60	1,484 (1.0%)	143,673 (99.0%)	1,360 (2.2%)	61,744 (97.8%)
50-59	1,255 (2.1%)^*^	59,925 (97.9%)	1,256 (4.9%)^*^	24,196 (95.1%)
40-49	1,010 (4.7%)^*^	20,667 (95.3%)	1,028 (6.9%)^*^	13,965 (93.1%)
< 40	333 (4.5%)^*^	7,107 (95.5%)	296 (4.5%)	6,218 (95.5%)
Ethnicity				
White	3,267 (1.8%)	180,070 (98.2%)	3,156 (3.7%)	83,026 (96.3%)
Black	162 (0.9%)^*^	17,201 (99.1%)	158 (2.2%)^*^	7,066 (97.8%)
Hispanic	321 (1.8%)	17,693 (98.2%)	289 (3.3%)^*^	8,588 (96.7%)
Asian	261 (2.1%)^*^	11,958 (97.9%)	265 (4.2%)^*^	5,995 (95.8%)
Others	71 (1.6%)^*^	4,450 (98.4%)	72 (4.7%)	1,448 (95.3%)
Marital status				
Single	965 (2.8%)	32,939 (97.2%)	961 (6.0%)	15,011 (94.0%)
Married	2,113 (1.7%)^*^	119,461 (98.3%)	2,078 (3.6%)^*^	54,915 (96.4%)
Others	1,004 (1.3%)^*^	78,892 (98.7%)	901 (2.4%)^*^	36,197 (97.6%)
Registry Area				
West	2,035 (1.7%)	118,951 (98.3%)	1,997 (3.5%)	55,049 (96.5%)
Central	929 (1.7%)	54,469 (98.3%)	886 (3.4%)	24,800 (96.5%)
East	1,118 (1.9%)	57,952 (98.1%)	1,057 (3.9%)^*^	26,274 (96.1%)
Year at diagnosis				
1973-1979	264 (1.3%)	20,607 (98.7%)	250 (2.9%)	8,488 (97.1%)
1980-1989	505 (1.8%)^*^	26,994 (98.2%)	475 (3.2%)	14,227 (96.8%)
1990-1999	692 (1.7%)^*^	38,875 (98.3%)	681 (3.3%)^*^	19,949 (96.7%)
2000-2009	1,835 (1.9%)^*^	96,275 (98.1%)	1,764 (3.8%)	45,258 (96.2%)
2010-2013	786 (1.6%)^*^	48,621 (98.4%)	770 (4.1%)	18,201 (95.5%)
Stage				
I	2,751 (1.8%)	147,820 (98.2%)	1,972 (8.5%)	21,201 (91.5%)
II	220 (2.2%)^*^	9,900 (97.8%)	547 (6.6%)	7,707 (93.4%)
III	329 (1.6%)	19,839 (98.4%)	878 (2.4%)^*^	35,963 (97.6%)
IV	242 (1.4%)	16,692 (98.6%)	424 (1.2%)^*^	35,310 (98.8%)
Unknown	540 (1.4%)^*^	37,121 (98.6%)	119 (2.0%)^*^	5,942 (98.0%)
Histology				
Endometrioid	3,185 (1.8%)	174,711 (98.2%)	2,069 (16.4%)	10,560 (83.6%)
Serous	281 (1.9%)^*^	14,144 (98.1%)	816 (1.5%)^*^	55,179 (98.5%)
Clear	32 (1.1%)	3,003 (98.9%)	148 (2.7%)^*^	5,392 (97.3%)
Others	584 (1.5%)	39,514 (98.5%)	907 (2.5%)^*^	34,992 (97.5%)
Grade				
1	1,535 (1.8%)	83,031 (98.2%)	950 (10.8%)	7,868 (89.2%)
2	1,183 (1.9%)^*^	61,203 (98.1%)	1,160 (7.0%)^*^	15,508 (93.0%)
3	631 (1.3%)	48,961 (98.7%)	913 (1.9%)^*^	45,913 (98.1%)
Unknown	733 (1.9%)^*^	38,177 (98.1%)	917 (2.4%)^*^	36,834 (97.6%)
Tumor size (cm)				
< 2.0 (or 10^†^)	451 (2.1%)	21,549 (98.0%)	1,383 (4.1%)	32,098 (95.9%)
≥ 2.0 (or 10^†^)	1,162 (1.5%)^*^	78,699 (98.5%)	948 (5.0%)^*^	18,179 (95.0%)
Unknown	2,469 (1.8%)	134,124 (98.2%)	1,609 (2.8%)^*^	55,846 (97.2%)

Number (%) per row or mean (±standard deviation) is shown (per column is shown in Supplementary Table 5). All covariates were statistically significant in chi-square test on univariable analysis. ^*^P < 0.05 on multivariable analysis with binary logistic regression models (entered all the listed covariates; the top item in each covariate served as the reference). ^†^size cutoff for the epithelial ovarian cancer cohort.

Histologic patterns of endometrial cancer and synchronous ovarian cancer were examined (Table 2). The most common histologic pattern was endometrioid types in the two cancer sites seen in nearly a half (45.6%), followed by endometrioid endometrial cancer with non-endometrioid ovarian cancer (33.0%) and non-endometrioid types in the two cancer sites (16.1%). Patient demographics and tumor characteristics significantly differ across the four histologic patterns (Table 2). Women with endometrial cancer were more likely to be young when the synchronous ovarian cancer was endometrioid type compared to non-endometrioid type (*P*<0.001). The proportion of women with endometrioid histology in the two cancer sites has significantly increased from 24.2% to 49.9% among the synchronous cancer cases during the study period (*P*<0.001). On the contrary, the proportion of endometrioid endometrial cancer and non-endometrioid ovarian cancer has significantly decreased during the same period (46.7% to 28.0%, *P*<0.001).

**Table 2:** Characteristics of endometrial cancer based on histology patterns of synchronous ovarian cancer

	Group 1	Group 2	Group 3	Group 4	*P*-value
Endometrial cancer	Endometrioid	Endometrioid	Non-endometrioid	Non-endometrioid	
Synchronous ovarian cancer	Endometrioid	Non-endometrioid	Endometrioid	Non-endometrioid	
Number	1,737 (45.6%)	1,258 (33.0%)	200 (5.2%)	615 (16.1%)	
Age (y)	51.8 (±11.2)	59.1 (±12.2)	52.2 (±11.2)	61.7 (±12.6)	**<0.001**
≥60	369 (27.2%)	586 (43.2%)	49 (3.6%)	352 (26.0%)	
50-59	574 (48.6%)	382 (32.3%)	67 (5.7%)	158 (13.4%)	
40-49	589 (61.7%)	228 (23.9%)	61 (6.4%)	77 (8.1%)	
< 40	205 (64.5%)	62 (19.5%)	23 (7.2%)	28 (8.8%)	
Ethnicity					**<0.001**
White	1,368 (44.7%)	1,042 (34.1%)	161 (5.3%)	487 (15.9%)	
Black	41 (28.1%)	55 (37.7%)	7 (4.8%)	43 (29.5%)	
Hispanic	146 (50.7%)	79 (27.4%)	16 (5.6%)	47 (16.3%)	
Asian	145 (58.7%)	62 (25.1%)	11 (4.5%)	29 (11.7%)	
Others	37 (52.1%)	20 (28.2%)	5 (7.0%)	9 (12.7%)	
Marital status					**<0.001**
Single	487 (53.6%)	261 (28.7%)	42 (4.6%)	118 (13.0%)	
Married	915 (45.9%)	655 (32.8%)	127 (6.4%)	298 (14.9%)	
Others	335 (36.9%)	342 (37.7%)	31 (3.4%)	199 (21.9%)	
Registry Area					**<0.001**
West	935 (49.0%)	586 (30.7%)	107 (5.6%)	279 (14.6%)	
Central	358 (41.5%)	294 (34.1%)	51 (5.9%)	159 (18.4%)	
East	444 (42.7%)	378 (36.3%)	42 (4.0%)	177 (17.0%)	
Year at diagnosis					**<0.001**
1973-1979	59 (24.2%)	114 (46.7%)	22 (9.0%)	49 (20.1%)	
1980-1989	156 (32.8%)	180 (37.9%)	49 (10.3%)	90 (18.9%)	
1990-1999	308 (47.2%)	219 (33.5%)	44 (6.7%)	82 (12.6%)	
2000-2009	847 (49.8%)	539 (31.7%)	57 (3.3%)	259 (15.2%)	
2010-2013	367 (49.9%)	206 (28.0%)	28 (3.8%)	135 (18.3%)	
Stage					**<0.001**
I	1,244 (48.2%)	870 (33.7%)	125 (4.8%)	342 (13.3%)	
II	111 (55.2%)	60 (29.9%)	7 (3.5%)	23 (11.4%)	
III	142 (46.3%)	90 (29.3%)	13 (4.2%)	62 (20.2%)	
IV	64 (29.5%)	61 (28.1%)	17 (7.8%)	75 (34.6%)	
Unknown	176 (34.9%)	177 (35.1%)	38 (7.5%)	113 (22.4%)	
Grade					**<0.001**
1	779 (53.8%)	504 (34.8%)	62 (4.3%)	102 (7.0%)	
2	578 (52.1%)	379 (34.1%)	55 (5.0%)	98 (8.8%)	
3	159 (27.2%)	180 (30.8%)	42 (7.2%)	204 (34.9%)	
Unknown	221 (33.1%)	195 (29.2%)	41 (6.1%)	211 (31.6%)	
Tumor size (cm)					**<0.001**
< 2.0	175 (41.4%)	156 (36.9%)	21 (5.0%)	71 (16.8%)	
≥ 2.0	581 (54.1%)	275 (25.6%)	53 (4.9%)	165 (15.4%)	
Unknown	981 (42.4%)	1,258 (35.8%)	200 (5.4%)	615 (16.4%)	

Combination patterns of endometrial cancer and synchronous epithelial ovarian cancer are displayed. Number (%) per row or mean (±standard deviation) are shown. Chi-square test for *P*-values. Significant *P*-values are emboldened.

**Figure 2:**
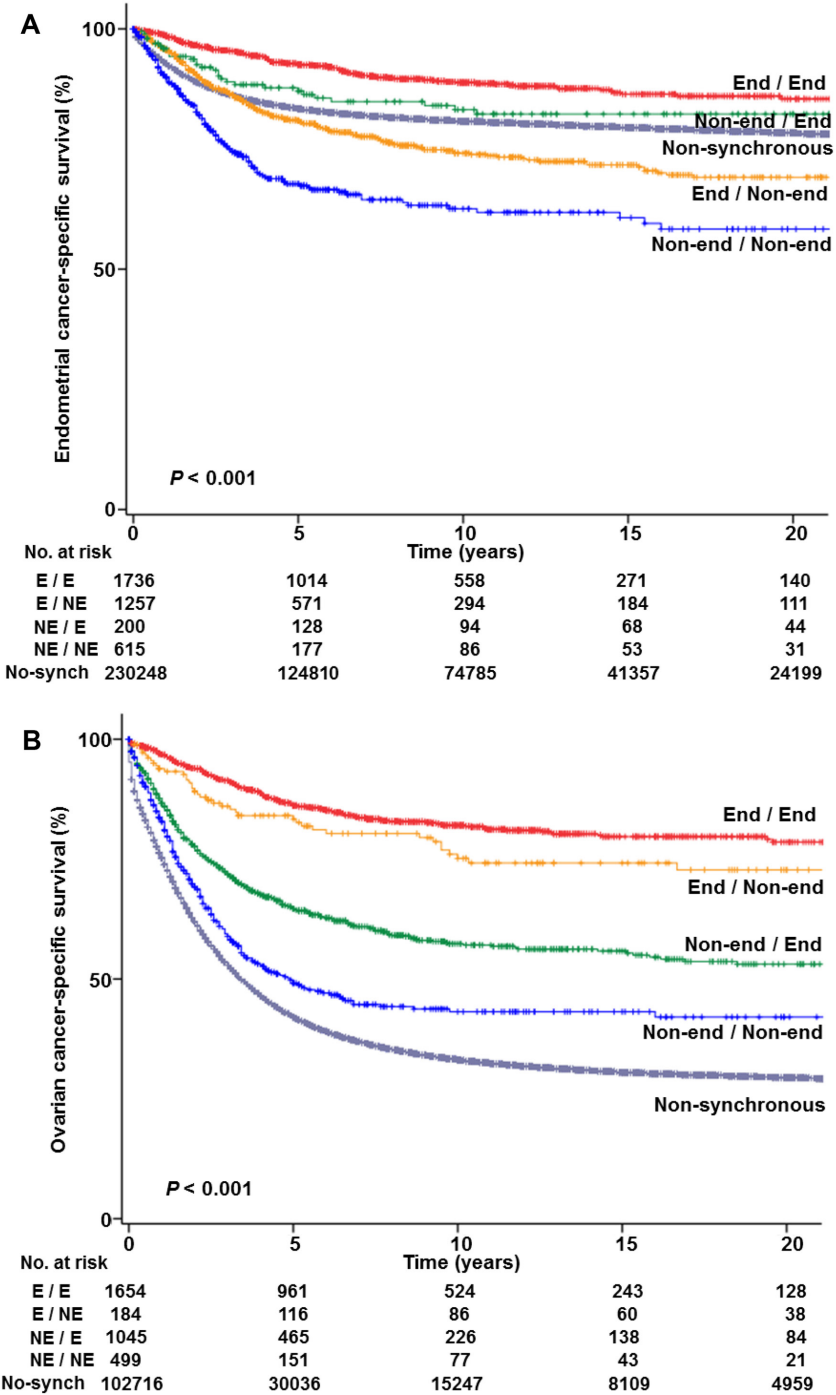
Cause-specific survival curves based on histologic patterns Log-rank test for *P*-values. **(A)** Endometrial cancer-specific survival in the endometrial cancer cohort and **(B)** ovarian cancer-specific survival in the epithelial ovarian cancer cohort are shown based on histology patterns in the two tumor sites (primary tumor site / synchronous tumor site; Supplementary Table 4). Abbreviations: End, endometrioid; and non-end, non-endometrioid.

Survival analysis was performed (Table 3). There were 40,056 deaths due to endometrial cancer in the study population. Median follow-up time among the cases without events was 7.5 years. Women with endometrial cancer who had synchronous ovarian cancer had a 10-year CSS similar to those who did not have synchronous ovarian cancer on univariable analysis (79.4% *versus* 80.7%, *P*=0.59). However, when stratified by histologic patterns in the two cancer sites, CSS significantly differed across the combination patterns (Figure 2A, *P*<0.001). That is, women whose tumors had endometrioid histology in the two cancer sites had a higher 10-year CSS compared to those without synchronous ovarian cancer (88.7% *versus* 80.7%, adjusted-hazard ratio [HR] 0.79, 95%CI 0.68-0.93, *P*=0.005).

**Table 3:** Multivariable analysis for cause-specific survival

	Endometrial cancer with synchronous ovarian cancer		Epithelial ovarian cancer with synchronous endometrial cancer	
	HR (95%CI)	*P*-value	HR (95%CI)	*P*-value
Age (y)				
< 40	1		1	
40-49	1.26 (1.14-1.39)	**<0.001**	1.51 (1.43-1.59)	**<0.001**
50-59	1.61 (1.47-1.76)	**<0.001**	1.82 (1.73-1.91)	**<0.001**
≥60	3.39 (3.10-3.71)	**<0.001**	2.77 (2.65-2.91)	**<0.001**
Ethnicity				
White	1		1	
Black	1.75 (1.69-1.80)	**<0.001**	1.21 (1.17-1.25)	**<0.001**
Hispanic	1.13 (1.09-1.18)	**<0.001**	0.97 (0.94-1.01)	0.11
Asian	0.97 (0.92-1.02)	0.26	0.91 (0.87-0.95)	**<0.001**
Others	1.06 (0.97-1.15)	0.20	0.96 (0.90-1.04)	0.34
Marital status				
Single	1		1	
Married	0.82 (0.80-0.85)	**<0.001**	0.86 (0.83-0.88)	**<0.001**
Others	1.16 (1.13-1.20)	**<0.001**	1.08 (1.05-1.11)	**<0.001**
Registry Area				
West	1		1	
Central	1.05 (1.03-1.08)	**0.011**	1.06 (1.04-1.08)	**<0.001**
East	1.03 (1.01-1.06)	**0.009**	0.94 (0.92-0.96)	**<0.001**
Year at diagnosis				
1973-1979	1		1	
1980-1989	1.28 (1.23-1.34)	**<0.001**	1.00 (0.96-1.03)	0.89
1990-1999	1.56 (1.49-1.63)	**<0.001**	1.05 (1.01-1.08)	**0.005**
2000-2009	1.58 (1.51-1.64)	**<0.001**	1.01 (0.98-1.04)	0.48
2010-2013	1.53 (1.45-1.61)	**<0.001**	0.84 (0.81-0.88)	**<0.001**
Stage				
I	1		1	
II	3.04 (2.90-3.19)	**<0.001**	2.57 (2.44-2.70)	**<0.001**
III	6.19 (5.99-6.39)	**<0.001**	5.73 (5.52-5.94)	**<0.001**
IV	19.4 (18.9-20.0)	**<0.001**	9.21 (8.88-9.54)	**<0.001**
Unknown	4.07 (3.95-4.21)	**<0.001**	6.23 (5.95-6.53)	**<0.001**
Tumor size (cm)				
< 2.0 (or 10^*^)	1		1	
≥ 2.0 (or 10^*^)	1.78 (1.68-1.89)	**<0.001**	0.94 (0.92-0.97)	**<0.001**
Unknown	1.86 (1.76-1.97)	**<0.001**	1.29 (1.26-1.31)	**<0.001**
Histology patterns^†^				
Non-synchronous	1		1	
Group 1 (E/E)	0.79 (0.68-0.93)	**0.005**	0.44 (0.39-0.50)	**<0.001**
Group 2 (E/NE)	1.58 (1.39-1.79)	**<0.001**	0.54 (0.39-0.73)	**<0.001**
Group 3 (NE/E)	0.94 (0.66-1.34)	0.73	0.78 (0.70-0.86)	**<0.001**
Group 4 (NE/NE)	1.57 (1.34-1.83)	**<0.001**	0.96 (0.85-1.10)	0.57

Cox proportional hazard regression models for cause-specific survival. All the listed covariates were entered in the final models. Significant *P*-values are emboldened. Unadjusted-HR is listed in Supplementary Table 6. ^*^cutoff for ovarian cancer. ^†^histology types for the primary tumor followed by the synchronous tumor are shown inside the brackets. Abbreviations: HR, hazard ratio; CI, confidence interval; E, endometrioid; and NE, non-endometrioid.

On the contrary, women with endometrial cancer who had synchronous non-endometrioid ovarian cancer had an increased endometrial cancer mortality compared to those without synchronous ovarian cancer: endometrial cancer with non-endometrioid ovarian cancer (10-year rates 74.2% *versus* 80.7%, adjusted-HR 1.58 95%CI 1.39-1.79, *P*<0.001) and non-endometrioid histology in the two cancer sites (62.9% *versus* 80.7%, adjusted-HR 1.57, 95%CI 1.34-1.83, *P*<0.001).

### Epithelial ovarian cancer cohort

Among 133,481 cases of ovarian malignancy cases in the database, metastatic tumors to the ovary from malignancy of non-ovarian origin (*n*=18,152), non-epithelial tumors (*n*=4,451), sarcoma (*n*=532), multiple diagnosis cases (duplicated or second entry, *n*=276), and unknown time interval due to missing data (*n*=7) were excluded. The remaining 110,063 cases represented primary epithelial ovarian cancer, and synchronous endometrial cancer was seen in 3,940 (3.6%, 95%CI 3.5-3.7) cases.

The proportion of epithelial ovarian cancer with synchronous endometrial cancer has significantly increased from 2.2% to 4.4% between 1973 and 2013 (APC 0.77, 95%CI 0.42-1.13, *P*<0.001, Figure 1A). The proportion of synchronous endometrial cancer cases among epithelial ovarian cancer increased between 31 and 48 years of age from 3.3% to 7.4% (APC 1.72, 95%CI 0.19-3.27, *P*=0.028) and then significantly decreased thereafter (7.4% at 48 years to 2.5% at 68 years, APC -5.52, 95%CI -6.27 to -4.76, *P*<0.001).

Characteristics of women with epithelial ovarian cancer who had synchronous endometrial cancer are shown in Table 1. Ovarian tumors associated with synchronous endometrial cancer were more likely to be stage I-II (incidence of synchronous endometrial cancer, 6.6-8.5%), of endometrioid histology (16.4%), and grade 1 tumors (10.8%) (all, adjusted-*P*<0.05). When synchronous cases were stratified by histologic patterns (Table 4), the proportion of women with endometrioid histology in the two cancer sites significantly increased during the study period (24.4% to 50.1%, *P*<0.001). Women were more likely to be older and have advanced-stage disease when tumors were of non-endometrioid histology in the two cancer sites (both, *P*<0.001).

**Table 4:** Characteristics of epithelial ovarian cancer based on histology patterns of synchronous endometrial cancer

	Group 1	Group 2	Group 3	Group 4	*P*-value
Ovarian cancer	Endometrioid	Endometrioid	Non-endometrioid	Non-endometrioid	
Synchronous endometrial cancer	Endometrioid	Non-endometrioid	Endometrioid	Non-endometrioid	
Number	1,847 (46.9%)	222 (5.6%)	1,227 (31.1%)	644 (16.3%)	
Age (y)	52.0 (±11.2)	53.2 (±11.8)	58.6 (±12.0)	61.3 (±12.0)	**<0.001**
≥60	393 (28.9%)	63 (4.6%)	547 (40.2%)	357 (26.3%)	
50-59	627 (49.9%)	65 (5.2%)	383 (30.5%)	181 (14.4%)	
40-49	630 (61.3%)	70 (6.8%)	243 (23.6%)	85 (8.3%)	
< 40	197 (66.6%)	24 (8.1%)	54 (18.2%)	21 (7.1%)	
Ethnicity					**<0.001**
White	1,473 (46.7%)	178 (5.6%)	1,007 (31.9%)	498 (15.8%)	
Black	47 (29.7%)	8 (5.1%)	54 (34.2%)	49 (31.0%)	
Hispanic	142 (49.1%)	20 (6.9%)	75 (26.0%)	52 (18.0%)	
Asian	148 (55.8%)	11 (4.2%)	72 (27.2%)	34 (12.8%)	
Others	37 (51.4%)	5 (6.9%)	19 (26.4%)	11 (15.3%)	
Marital status					**<0.001**
Single	519 (54.0%)	47 (4.9%)	266 (27.7%)	129 (13.4%)	
Married	973 (46.8%)	135 (6.5%)	652 (31.4%)	318 (15.3%)	
Others	355 (39.4%)	40 (4.4%)	309 (34.3%)	197 (21.9%)	
Registry Area					**<0.001**
West	998 (50.0%)	122 (6.1%)	562 (28.1%)	315 (15.8%)	
Central	384 (43.3%)	52 (5.9%)	289 (32.6%)	161 (18.2%)	
East	465 (44.0%)	48 (4.5%)	376 (35.6%)	168 (15.9%)	
Year at diagnosis					**<0.001**
1973-1979	61 (24.4%)	25 (10.0%)	110 (44.0%)	54 (21.6%)	
1980-1989	165 (34.7%)	48 (10.1%)	173 (36.4%)	89 (18.7%)	
1990-1999	334 (49.0%)	50 (7.3%)	203 (29.8%)	94 (13.8%)	
2000-2009	901 (51.1%)	64 (3.6%)	534 (30.3%)	265 (15.0%)	
2010-2013	386 (50.1%)	35 (4.5%)	207 (26.9%)	142 (18.4%)	
Stage					**<0.001**
I	1,132 (57.4%)	125 (6.3%)	506 (25.7%)	209 (10.6%)	
II	295 (53.9%)	29 (5.3%)	155 (28.3%)	68 (12.4%)	
III	284 (32.3%)	45 (5.1%)	341 (38.8%)	208 (23.7%)	
IV	102 (24.1%)	20 (4.7%)	176 (41.5%)	126 (29.7%)	
Unknown	34 (28.6%)	3 (2.5%)	49 (41.2%)	33 (27.7%)	
Grade					**<0.001**
1	604 (63.6%)	69 (7.3%)	191 (20.1%)	86 (9.1%)	
2	688 (59.3%)	69 (5.9%)	284 (24.5%)	119 (10.3%)	
3	261 (28.6%)	30 (3.3%)	379 (41.5%)	243 (26.6%)	
Unknown	294 (32.1%)	54 (5.9%)	373 (40.7%)	196 (21.4%)	
Tumor size (cm)					**<0.001**
< 10	640 (46.3%)	83 (6.0%)	417 (30.2%)	243 (17.6%)	
≥ 10	550 (58.0%)	43 (4.5%)	247 (26.1%)	108 (11.4%)	
Unknown	657 (40.8%)	96 (6.0%)	563 (35.0%)	293 (18.2%)	

Combination patterns of epithelial ovarian cancer and synchronous endometrial cancer are displayed. Number (%) per row or mean (±standard deviation) are shown. Chi-square test for *P*-values. Significant *P*-values are emboldened.

In this cohort, there were 60,798 women who died of epithelial ovarian cancer, and the median follow-up time was 5.7 years for women without events. Women with ovarian cancer who had synchronous endometrial cancer had a significantly improved 10-year CSS compared to those without synchronous endometrial cancer (68.3% *versus* 33.1%, *P*<0.001). When histology patterns were stratified (Figure 2B), regardless of histology type in the endometrial tumor, women whose epithelial ovarian cancer had endometrioid histology had nearly a 50% reduction in ovarian cancer mortality compared to non-synchronous cancer cases on multivariable analysis (endometrioid ovarian and endometrioid endometrial cancers, 81.9% *versus* 33.1%, adjusted-HR 0.44, 95%CI 0.39-0.50, *P*<0.001; and endometrioid ovarian and non-endometrioid endometrial cancers, 76.0% *versus* 33.1%, adjusted-HR 0.54, 95%CI 0.39-0.73, *P*<0.001). Similarly, if endometrial cancer is endometrioid type, regardless of ovarian cancer histology type, SEOC was associate with improved CSS compared to non-synchronous ovarian cancer (adjusted-*P*<0.001).

## DISCUSSION

The main findings of this study are that there were significant changes in trends and characteristics of SEOC in the past decades and that the histologic pattern of endometrial and ovarian cancers impacts survival. Moreover, this study found that the peak age at diagnosis of SEOC was in the late-40’s.

In our study, there was a paradoxical change in trends of endometrial cancer with synchronous ovarian cancer (decreasing) and epithelial ovarian cancer with synchronous endometrial cancer (increasing). One speculation for these trends is the demographic changes in these two cancers in the United States: decreasing incidence of ovarian cancer and increasing incidence of endometrial cancer [8–12]. Assuming that the incidence of SEOC is constant, the relative proportion of SEOC will be decreased if incidence of endometrial cancer is increased. Conversely, if incidence of ovarian cancer is decreased, the relative proportion of SEOC will be increased.

Another speculation for these changes in trends of SEOC may be an impact of the diagnostic criteria. The reflection point of the proportion of women with endometrial cancer who had synchronous ovarian cancer was in the mid-1980s, and the proportion has gradually decreased after the point (Figure 1A). This is the time at which the landmark study for the diagnostic criteria of SEOC was reported and the criteria were endorsed in subsequent years [5–7]. One may speculate that these publications may have impacted these temporal trend changes.

Prior studies have reported that 66-86% of SEOC have concordant endometrioid histology in the two cancer sites [1, 7]. Moreover, endometrioid endometrial cancer is significantly associated with increased risk of secondary primary ovarian cancer with endometrioid histology [27]. Our population-based study showed that concordant endometrioid tumors in the endometrium and the ovary were seen in nearly half of the synchronous cases thereby endorsing these previous studies. An interesting observation in our study is that the proportion of concordant endometrioid tumors in the two cancer sites among SEOC has been increasing over the time. While the exact causality is unknown, it is possible that the current approach to diagnose SEOC, relying largely upon histopathology characteristics, may have a certain limitation [28].

Specifically, distinguishing SEOC from metastatic ovarian cancer to the endometrium (or metastatic endometrial cancer to the ovary) has long been challenging, especially if the histologic type is concordant in the two cancer sites such as endometrioid tumors [29–31]. Recent studies have shown that the concordance rate between histopathologic diagnosis and molecular diagnosis was considerably low, and it may be possible that the cases that met the diagnostic criteria for SEOC were actually metastatic endometrial cancer to the ovary or *vice versa* [29–31]. Integrating molecular diagnosis to differentiate synchronous *versus* metastatic cases would be useful in the proper diagnosis of SEOC [32, 33].

SEOC has been thought to be a disease of young women [4]. Our results are more specific to show that the proportion of SEOC increased to the late-40s and then decreased thereafter (Figure 1B), highlighting that SEOC is indeed a disease of middle-aged women. One hypothesis to link this association is the possibility of Lynch syndrome. The mean age of endometrial cancer diagnosis in women with Lynch syndrome is in the late-40s (47-49 years) and the mean age of ovarian cancer in women with Lynch syndrome is also in the 40s (42-49 years) [34]. Moreover, the incidence of synchronous endometrial cancer in ovarian cancer among women with Lynch syndrome is reported as 21.5% [35]. Collectively, genetic assessment and testing is highly recommended for women with SEOC.

The clinical outcome of women with SEOC seems inconsistent between studies. Some studies concluded that women with SEOC have a favorable prognosis while others have concluded that no difference in prognosis to non-synchronous endometrial or ovarian cancer [1, 7, 36]. Because these previous studies were either lacking a control group or were conducted with limited sample sizes, our study is more informative in that survival of SEOC largely depends on the histologies of the two cancer sites.

There are various strengths of this study. First, this is a population-based study linking multiple datasets for endometrial and ovarian cancers by examining the SEER Program. Second, this study examined nearly four decades of cases to evaluate time trends of synchronous tumors, providing clinically meaningful information regarding this disease entity. Last, histology pattern was examined in both endometrial and ovarian cancer in this study. Limitations of this study include the fact that central pathology review was not performed to confirm the diagnosis of SEOC *versus* metastatic tumors from endometrial or ovarian cancer. As described above, lack of central pathology review including clonality analysis results in possible over-diagnosis of SEOC. Information for molecular clonality analysis was not available in this database. In addition, this database does not have genetic information available. Thus, it remains unknown what percentage of women with SEOC had Lynch syndrome. This study was conducted only for a US population; thus, generalizability or our results in other population remains unknown and merits further study.

A weakness of the study is the arbitrary cutoff used to define the SEOC. One may be concerned that the cutoff of a 4-month time difference between the two cancer diagnoses may be too liberal. However, we have chosen this cutoff based on a theoretical rationale described earlier. In a post-hoc analysis, we examined a stricter cutoff of a 2-month time difference between the two cancer diagnoses. Similar results were re-demonstrated for SEOC trends per calendar year and patient age (Supplementary Figure 2A-2B).

Last, survival analysis in SEOC would be challenging in various aspects. First, if the histology types of the two cancer sites are concordant, it will be likely difficult to determine the actual attribution of cause of death, particularly when both cancers are early-stage. Second, when the patient has a short follow-up time, it will be unknown whether the patient will develop a secondary primary malignancy [37]. In our study, we used a fairly short cutoff of 4-month interval for defining SEOC and thus this time-lead bias would be most likely minimized.

A clinical implication of our results is in the area of surgical management and planning. First, while supracervical hysterectomy is suggested as an alternative mode of hysterectomy in the management of women with epithelial ovarian cancer [38], it will be reasonable to perform total hysterectomy given that considerable fractions of women with epithelial ovarian cancer may have a concurrent endometrial cancer. If supracervical hysterectomy is performed, tumor cells from the endometrial cancer left in the cervical stump or the cervix can be the source of residual tumors resulting in decreased survival outcome [39]. Therefore, if supracervical hysterectomy for ovarian cancer is planned, preoperative assessment of endometrial pathology may be a reasonable approach to rule out synchronous endometrial cancer. Similarly, when fertility preservation is considered for young women with apparent stage I epithelial ovarian cancer, evaluation of endometrial pathology is an important preoperative assessment to rule out synchronous endometrial cancer [40].

Second, if young women with clinically early-stage low-grade endometrial cancer desire future fertility with a non-surgical approach, evaluation of adnexal pathology is necessary as a routine pretreatment evaluation given that younger reproductive age women have higher risk of synchronous ovarian cancer. If imaging or biomarker testing suspects the presence of synchronous ovarian cancer, non-surgical approaches would not be advisable. Similarly, a thorough and careful intraoperative assessment of the adnexa is recommended for young women who desire ovarian preservation for clinical early-stage low-grade endometrial cancer [24].

## SUPPLEMENTARY MATERIALS FIGURES AND TABLES


