# Supplementary material for: Trends and outcomes of women with synchronous endometrial and ovarian cancer
Source: Oncotarget. 2018 Jun 19;9(47):28757–71. doi: 10.18632/oncotarget.25550 (PMC6033337; doi:10.18632/oncotarget.25550)
Supplement: Supplementary file 1 [file oncotarget-09-28757-s001.pdf]

Trends and outcomes of women with synchronous endometrial and ovarian cancer

SUPPLEMENTARY MATERIALS

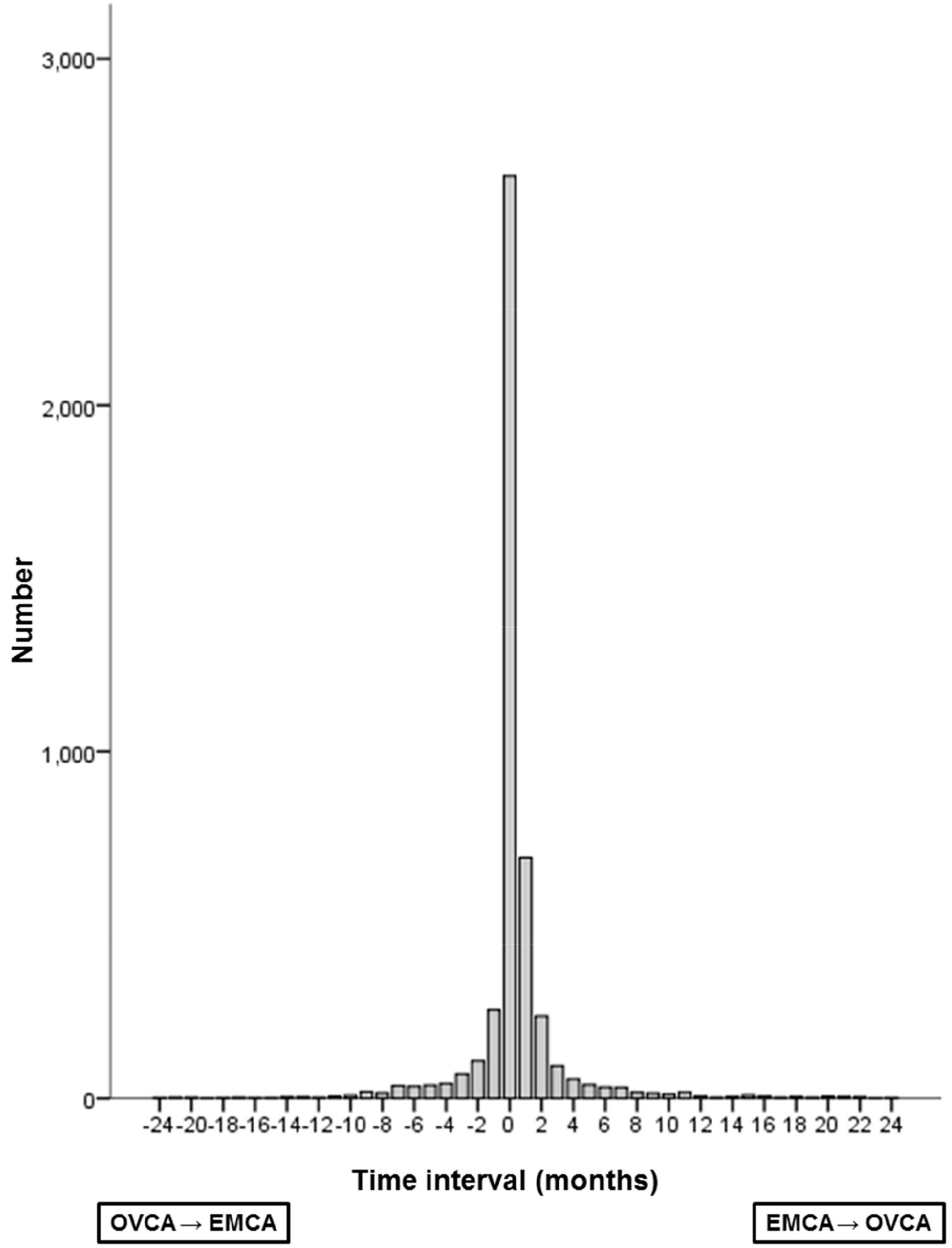

Supplementary Figure 1: Time interval for secondary primary cancers. Time interval between ovarian cancer and endometrial cancer is shown. Abbreviations: EMCA, endometrial cancer; and OVCA, ovarian cancer.

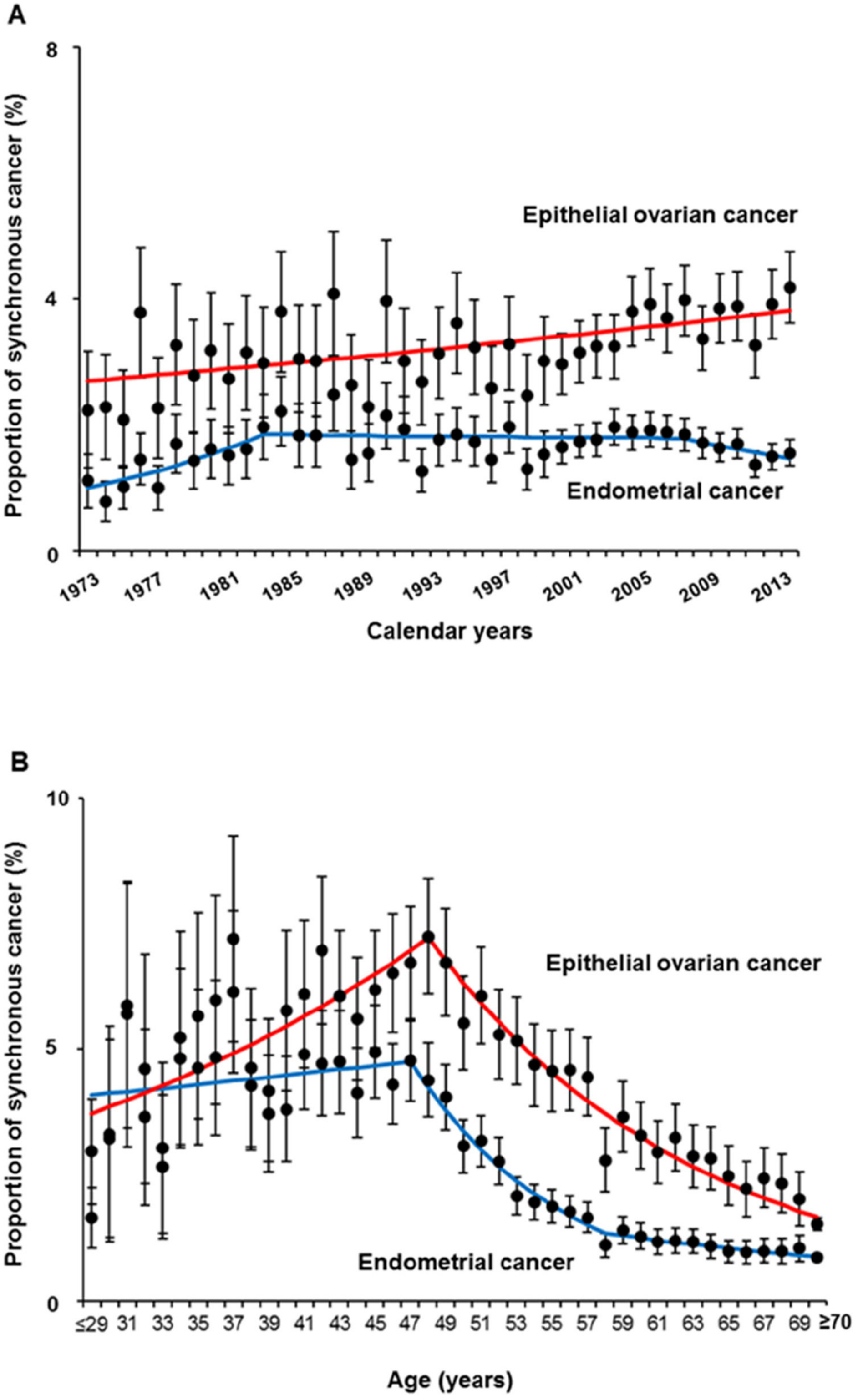

Supplementary Figure 2: Trends of synchronous endometrial and ovarian cancer (cutoff 2 months). Proportion of synchronous cancer is shown per **(A)** calendar year and **(B)** age. Blue line depicts a trend of proportion of women with endometrial cancer who had synchronous ovarian cancer. Red line depicts a trend of proportion of women with epithelial ovarian cancer who had synchronous endometrial cancer. Dots represent percent proportion and error bars represent 95% confidence interval.

Supplementary Table 1: List of histologic subtype codes (endometrial cancer cohort)

Endometrioid: 8140-3, 8262-3, 8380-3, 8382-3, 8383-3.
Serous: 8260-3, 8441-3, 8450-3, 8460-3,8461-3.
Clear cell: 8005-3, 8310-3,
Others: 8000-3, 8001-3, 8002-3, 8003-3, 8010-3, 8011-3, 8012-3, 8013-3, 8014-3, 8015-3, 8022-3, 8030-3, 8031-3,8032-3,8033-3,8040-3,8041-3,8045-3,8046-3,8050-3,88050, 8090-3,8098-3,8120-3,8123-3,8130-3,8143-3,8144-3,8145-3,8200-3,8201-3,8210-3,8211-3,8220-3,8221-3,8230-3,8244-3,8246-3,8249-3, 8261-3, 8263-3,8290-3, 8313-3,8320-3, 8340-3,8370-3, 8381-3, 8384-3,8410-3,8440-3, 8510-3,8550-3, 8562-3, 8571-3,8572-3,8573-3,8574-3,8575-3,8576-3,8830-3, 8960-3, 8990-3, 9014-3,9015-3, 9130-3,9133-3,9150-3, 9508-3.
Carcinosarcoma: 8950-3, 8951-3, 8980-3, 8981-3,
Sarcoma: 8004-3, 8800-3,8801-3,8802-3,8803-3,8804-3,8805-3,8810-3,8811-3,8814-3,8840-3,8850-3, 8853-3,8854-3,8855-3,8858-3, 8860-3,8890-3,8891-3,8895-3,8896-3,8897-3,8900-3,8901-3,8902-3,8910-3,8912-3,8920-3,8930-3,8931-3,8933-3,8934-3,8935-3, 8963-3, 8991-3, 9120-3, 9180-3,9220-3,9240-3,9260-3, 9581-3
Metastatic tumor: 8141-3, 8490-3, 8580-3,8590-3,8600-3,8620-3,8650-3,8680-3,8720-3, 8936-3, 9064-3,9071-3,9080-3,9085-3,9100-3,9101-3,9104-3,9105-3,9110-3, 9364-3,9380-3,9473-3, 9560-3,9561-3
Undifferentiated: 8020-3, 8021-3
Squamous: 8051-3,8052-3,8070-3,8071-3,8072-3,8073-3,8074-3,8075-3,8076-3,8083-3,8084-3
Mixed: 8255-3, 8323-3, 8940-3
Mucinous: 8470-3, 8471-3, 8480-3, 8481-3, 8482-3
Adenosquamous: 8560-3
Adenocarcinoma with squamous metaplasia: 8570-3

Supplementary Table 2: List of histologic subtype codes (epithelial ovarian cancer cohort)

Endometrioid: 8380-3, 8381-3, 8382-3, 8383-3.
Serous: 8050-3, 8260-3, 8341-3, 8441-3, 8442-3, 8450-3, 8460-3, 8461-3, 8462-3, 8463-3.
Clear cell: 8005-3, 8310-3, 8443-3, 8444-3
Mucinous cell: 8470-3, 8471-3, 8472-3, 8480-3, 8481-3, 8482-3
Mixed adenocarcinoma: 8255-3, 8323-3, 8940-3, 8950-3
Other EOC: 8010-3, 8011-3, 8020-3, 8021-3, 8120-3, 8122-3, 8130-3, 8140-3, 8200-3, 8201-3, 8230-3, 8231-3, 8313-3, 8440-3, 8570-3, 8572-3, 8574-3, 8575-3, 9000-3, 9013-3, 9014-3, 9015-3
Germ cell tumors: 8052-3, 8070-3, 8071-3, 8072-3, 8074-3, 8084-3, 8243-3, 8244-3, 8245-3, 8246-3, 8249-3, 8390-3, 8401-4,8410-3, 8439-3, 8560-3, 9060-3, 9063-3, 9064-3, 9065-3, 9070-3, 9071-3, 9072-3, 9073-3, 9080-3, 9081-3, 9082-3, 9083-3, 9084-3, 9085-3, 9090-3, 9091-3, 9100-3, 9101-3, 9105-3, 9110-3, 9364-3, 9391-3, 9392-3, 9400-3, 9460-3, 9490-3, 9500-3
Sex cord-stromal tumors: 8032-3, 8320-3, 8590-3, 8593-3, 8600-3, 8620-3, 8621-3, 8622-3, 8623-3, 8630-3, 8631-1, 8632-3, 8634-3, 8640-3, 8650-3, 8670-3, 9580-3
Carcinosarcoma: 8951-3, 8980-3, 8981-3
Sarcoma: 8004-3, 8800-3, 8801-3, 8802-3, 8803-3, 8804-3, 8805-3, 8810-3, 8840-3, 8850-3, 8851-3, 8854-3, 8890-3,8891-3, 8896-3, 8900-3, 8901-3, 8910-3, 8920-3, 8921-3, 8930-3, 8931-3, 8933-3, 8935-3, 8936-3, 8963-3, 8990-3, 9044-3, 9120-3, 9180-3, 9220-3, 9260-3, 9473-3
Other primary tumors: 8000-3, 8001-3, 8002-3, 8003-3, 8012-3, 8013-3, 8030-3, 8031-3, 8033-3, 8040-3, 8041-3, 8043-3, 8045-3, 8046-3, 8100-3, 8123-3, 8240-3, 8330-3, 8331-3, 8335-3, 8337-3, 8340-3, 8344-3, 8452-3, 8562-3, 8576-3, 8806-3, 8830-3
Metastatic tumor: 8015-3, 8022-3, 8141-3, 8144-3, 8250-3, 8251-3, 8262-3, 8263-3, 8290-3, 8312-3, 8384-3, 8490-3, 8500-3, 8503-3, 8504-3, 8507-3, 8510-3, 8523-3, 8542-3, 8550-3, 8720-3, 8740-3

Supplementary Table 3: Frequencies of SEOC per age

Age	Endometrial cancer cohort				Epithelial ovarian cancer cohort			
	SEOC	(%) per age	(%) per SEOC	Total	SEOC	(%) per age	(%) per SEOC	Total
≤29	31	3.1	0.8	1,010	31	1.8	0.8	1,744
30	10	3.2	0.2	310	9	3.3	0.2	271
31	21	5.9	0.5	358	17	5.7	0.4	298
32	16	3.7	0.4	438	17	5.2	0.4	325
33	13	2.7	0.3	487	12	3.0	0.3	395
34	28	5.0	0.7	560	24	5.7	0.6	421
35	35	4.9	0.9	711	29	5.9	0.7	494
36	38	5.1	0.9	745	34	6.8	0.9	501
37	58	6.8	1.4	847	47	7.7	1.2	611
38	42	4.4	1.0	955	38	5.5	1.0	689
39	41	4.0	1.0	1,019	38	5.0	1.0	765
40	51	4.0	1.2	1,286	51	6.1	1.3	831
41	73	5.1	1.8	1,445	65	6.4	1.6	1,017
42	80	5.1	2.0	1,566	83	7.1	2.1	1,161
43	80	4.8	2.0	1,661	85	6.5	2.2	1,303
44	81	4.3	2.0	1,885	83	6.0	2.1	1,372
45	112	5.3	2.7	2,121	108	6.6	2.7	1,631
46	113	4.6	2.8	2,483	118	7.0	3.0	1,674
47	137	5.0	3.4	2,745	138	7.1	3.5	1,934
48	135	4.5	3.3	3,027	144	7.4	3.7	1,946
49	148	4.3	3.6	3,458	153	7.2	3.9	2,124
50	133	3.3	3.3	4,065	140	6.1	3.6	2,299
51	154	3.3	3.8	4,622	154	6.5	3.9	2,355
52	144	2.9	3.5	4,959	134	5.6	3.4	2,399
53	125	2.2	3.1	5,628	133	5.3	3.4	2,493
54	124	2.0	3.0	6,093	128	5.0	3.2	2,558
55	127	1.9	3.1	6,637	126	4.8	3.2	2,623
56	126	1.8	3.1	6,931	123	4.8	3.1	2,569
57	126	1.7	3.1	7,387	130	4.8	3.3	2,694
58	84	1.2	2.1	7,270	81	3.0	2.1	2,676
59	112	1.5	2.7	7,588	107	3.8	2.7	2,786
60	105	1.4	2.6	7,742	100	3.5	2.5	2,821
61	93	1.2	2.3	7,709	89	3.1	2.3	2,833
62	99	1.3	2.4	7,723	95	3.4	2.4	2,764
63	92	1.2	2.3	7,564	87	3.1	2.2	2,820
64	83	1.2	2.0	7,212	80	2.9	2.0	2,748
65	76	1.0	1.9	7,605	72	2.6	1.8	2,731
66	69	1.0	1.7	6,896	65	2.4	1.6	2,737
67	71	1.0	1.7	6,805	71	2.7	1.8	2,616
68	67	1.0	1.6	6,429	65	2.5	1.6	2,647
69	64	1.0	1.6	6,105	54	2.1	1.4	2,625
≥70	665	0.9	16.3	73,367	582	1.6	14.8	35,762
Total	4,082	1.7	100.0	235,454	3,940	3.6	100.0	110,063

Abbreviation: SEOC, synchronous endometrial and ovarian cancer.

Supplementary Table 4: Combination patterns of endometrial and ovarian cancers

Endometrial cancer cohort		
	**Endometrial cancer**	**synchronous ovarian cancer**
Group 1 (E/E)	Endometrioid	Endometrioid
Group 2 (E/NE)	Endometrioid	Non-endometrioid
Group 3 (NE/E)	Non-endometrioid	Endometrioid
Group 4 (NE/NE)	Non-endometrioid	Non-endometrioid
**Epithelial ovarian cancer cohort**		
	**Epithelial ovarian cancer**	**synchronous endometrial cancer**
Group 1 (E/E)	Endometrioid	Endometrioid
Group 2 (E/NE)	Endometrioid	Non-endometrioid
Group 3 (NE/E)	Non-endometrioid	Endometrioid
Group 4 (NE/NE)	Non-endometrioid	Non-endometrioid

Abbreviations: E, endometrioid; and NE, non-endometrioid.

Supplementary Table 5: Patient demographics

	Endometrial cancer cohort		Epithelial ovarian cancer cohort	
**Characteristic**	**Synchronous ovarian cancer (+)**	**Synchronous ovarian cancer (-)**	**Synchronous endometrial cancer (+)**	**Synchronous endometrial cancer (-)**
Number	4,082 (1.7%)	231,372 (98.3%)	3,940 (3.6%)	106,123 (96.4%)
Age (y)	56.1 (±12.6)	63.5 (±12.4)	55.6 (±12.2)	62.3 (±14.3)
≥60	1,484 (36.4%)	143,673 (62.1%)	1,360 (34.5%)	61,744 (58.2%)
50-59	1,255 (30.7%)*	59,925 (25.9%)	1,256 (31.9%)*	24,196 (22.8%)
40-49	1,010 (24.7%)*	20,667 (8.9%)	1,028 (26.1%)*	13,965 (13.2%)
< 40	333 (8.2%)*	7,107 (3.1%)	296 (7.5%)	6,218 (5.9%)
Ethnicity				
White	3,267 (80.0%)	180,070 (77.8%)	3,156 (80.1%)	83,026 (78.2%)
Black	162 (4.0%)*	17,201 (7.4%)	158 (4.0%)*	7,066 (6.7%)
Hispanic	321 (7.9%)	17,693 (7.6%)	289 (7.3%)*	8,588 (8.1%)
Asian	261 (6.4%)*	11,958 (5.2%)	265 (6.7%)*	5,995 (5.6%)
Others	71 (1.7%)*	4,450 (1.9%)	72 (1.8%)	1,448 (1.4%)
Marital status				
Single	965 (23.6%)	32,939 (14.2%)	961 (6.0%)	15,011 (94.0%)
Married	2,113 (51.8%)*	119,461 (51.6%)	2,078 (3.6%)*	54,915 (96.4%)
Others	1,004 (24.6%)*	78,892 (34.1%)	901 (2.4%)*	36,197 (97.6%)
Registry Area				
West	2,035 (49.9%)	118,951 (51.4%)	1,997 (50.7%)	55,049 (51.9%)
Central	929 (22.8%)	54,469 (23.5%)	886 (22.5%)	24,800 (23.4%)
East	1,118 (27.4%)	57,952 (25.0%)	1,057 (26.8%)*	26,274 (24.8%)
Year at diagnosis				
1973-1979	264 (6.5%)	20,607 (8.9%)	250 (6.3%)	8,488 (8.0%)
1980-1989	505 (12.4%)*	26,994 (11.7%)	475 (12.1%)	14,227 (13.4%)
1990-1999	692 (17.0%)*	38,875 (16.8%)	681 (17.3%)*	19,949 (18.8%)
2000-2009	1,835 (45.0%)*	96,275 (41.6%)	1,764 (44.8%)	45,258 (42.6%)
2010-2013	786 (19.3%)*	48,621 (21.0%)	770 (19.5%)	18,201 (17.2%)
Stage				
I	2,751 (67.4%)	147,820 (63.9%)	1,972 (50.1%)	21,201 (20.0%)
II	220 (5.4%)*	9,900 (4.3%)	547 (13.9%)	7,707 (7.3%)
III	329 (8.1%)	19,839 (8.6%)	878 (22.3%)*	35,963 (33.9%)
IV	242 (5.9%)	16,692 (7.2%)	424 (10.8%)*	35,310 (33.3%)
Unknown	540 (13.2%)*	37,121 (16.0%)	119 (3.0%)*	5,942 (5.6%)
Histology				
Endometrioid	3,185 (78.0%)	174,711 (75.5%)	2,069 (52.5%)	10,560 (10.0%)
Serous	281 (6.9%)*	14,144 (6.1%)	816 (20.7%)*	55,179 (52.0%)
Clear	32 (0.8%)	3,003 (1.3%)	148 (3.8%)*	5,392 (5.1%)
Others	584 (14.3%)	39,514 (17.1%)	907 (23.0%)*	34,992 (33.0%)
Grade				
1	1,535 (37.6%)	83,031 (35.9%)	950 (24.1%)	7,868 (7.4%)
2	1,183 (29.0%)*	61,203 (26.5%)	1,160 (29.4%)*	15,508 (14.6%)
3	631 (15.5%)	48,961 (21.2%)	913 (23.2%)*	45,913 (43.3%)
Unknown	733 (18.0%)*	38,177 (16.5%)	917 (23.3%)*	36,834 (34.7%)
Tumor size (cm)				
< 2.0 (or 10†)	451 (11.0%)	21,549 (9.3%)	1,383 (35.1%)	32,098 (30.2%)
≥ 2.0 (or 10†)	1,162 (28.5%)*	78,699 (32.7%)	948 (24.1%)*	18,179 (17.1%)
Unknown	2,469 (60.5%)	134,124 (58.0%)	1,609 (40.8%)*	55,846 (52.6%)

Number (%) per column or mean (±standard deviation) is shown. All covariates were statistically significant in chi-square test on univariable analysis. *$P < 0.05$ on multivariable analysis with binary logistic regression models (entered all the listed covariates; the top item in each covariate served as the reference). †size cutoff for the epithelial ovarian cancer cohort.

Supplementary Table 6: Univariable analysis for cause-specific survival

	Endometrial cancer with synchronous ovarian cancer		Epithelial ovarian cancer with synchronous endometrial cancer	
	**HR (95%CI)**	***P*-value**	**HR (95%CI)**	***P*-value**
Age (y)				
< 40	1		1	
40-49	1.22 (1.11-1.35)	**<0.001**	1.66 (1.58-1.75)	**<0.001**
50-59	1.62 (1.48-1.78)	**<0.001**	2.22 (2.12-2.33)	**<0.001**
≥60	3.82 (3.50-4.18)	**<0.001**	4.08 (3.89-4.27)	**<0.001**
Ethnicity				
White	1		1	
Black	2.52 (2.45-2.60)	**<0.001**	1.27 (1.23-1.31)	**<0.001**
Hispanic	1.07 (1.03-1.12)	**<0.001**	0.83 (0.81-0.86)	**<0.001**
Asian	0.87 (0.83-0.91)	**<0.001**	0.66 (0.64-0.69)	**<0.001**
Others	0.88 (0.81-0.96)	**0.004**	0.80 (0.75-0.87)	**<0.001**
Marital status				
Single	1		1	
Married	0.75 (0.73-0.78)	**<0.001**	1.11 (1.08-1.13)	**<0.001**
Others	1.44 (1.39-1.48)	**<0.001**	1.70 (1.65-1.74)	**<0.001**
Registry Area				
West	1		1	
Central	1.12 (1.09-1.15)	**<0.001**	1.13 (1.10-1.15)	**<0.001**
East	1.16 (1.13-1.18)	**<0.001**	1.02 (0.99-1.04)	0.086
Year at diagnosis				
1973-1979	1		1	
1980-1989	1.27 (1.22-1.32)	**<0.001**	0.97 (0.94-1.01)	0.10
1990-1999	1.21 (1.16-1.26)	**<0.001**	0.91 (0.88-0.94)	**<0.001**
2000-2009	1.27 (1.23-1.32)	**<0.001**	0.83 (0.81-0.86)	**<0.001**
2010-2013	1.23 (1.18-1.29)	**<0.001**	0.66 (0.64-0.69)	**<0.001**
Stage				
I	1		1	
II	3.46 (3.30-3.63)	**<0.001**	2.77 (2.63-2.91)	**<0.001**
III	6.80 (6.59-7.02)	**<0.001**	6.54 (6.31-6.78)	**<0.001**
IV	22.3 (21.7-22.9)	**<0.001**	11.5 (11.1-11.9)	**<0.001**
Unknown	3.83 (3.74-3.94)	**<0.001**	7.91 (7.55-8.28)	**<0.001**
Tumor size (cm)				
< 2.0 (or 10*)	1		1	
≥ 2.0 (or 10*)	3.48 (3.28-3.68)	**<0.001**	0.73 (0.71-0.75)	**<0.001**
Unknown	3.00 (2.83-3.17)	**<0.001**	1.47 (1.45-1.50)	**<0.001**
Histology patterns†				
Non-synchronous	1		1	
Group 1 (E/E)	0.49 (0.42-0.58)	**<0.001**	0.18 (0.16-0.20)	**<0.001**
Group 2 (E/NE)	1.24 (1.10-1.41)	**0.001**	0.25 (0.19-0.34)	**<0.001**
Group 3 (NE/E)	0.80 (0.58-1.14)	0.22	0.51 (0.46-0.56)	**<0.001**
Group 4 (NE/NE)	1.95 (1.67-2.28)	**<0.001**	0.76 (0.67-0.87)	**<0.001**

Cox proportional hazard regression models for cause-specific survival. Significant *P*-values are emboldened. *cutoff for ovarian cancer. †histology types for the primary tumor followed by the synchronous tumor are shown inside the brackets. Abbreviations: HR, hazard ratio; CI, confidence interval; E, endometrioid; and NE, non-endometrioid.
